# Exploring Prescription Practices: Insights from an Antimicrobial Stewardship Program at a Tertiary Healthcare Facility, Rwanda

**DOI:** 10.3390/antibiotics13060548

**Published:** 2024-06-12

**Authors:** Misbah Gashegu, Noel Gahamanyi, François Xavier Ndayambaje, Jean Bosco Munyemana, Vedaste Ndahindwa, Fredrick Lukwago, Lambert Ingabire, Fiona Gambanga, Pierre Gashema, Albert Tuyishime, Tafadzwa Dzinamarira, Damas Dukundane, Thierry Zawadi Muvunyi, Claude Mambo Muvunyi

**Affiliations:** 1 Rwanda Biomedical Centre, Kigali P.O. Box 7162, Rwanda; misbah.gashegu@rbc.gov.rw (M.G.); noel.gahamanyi@rbc.gov.rw (N.G.); albert.tuyishime@rbc.gov.rw (A.T.); 2 Biology Department, College of Science and Technology, University of Rwanda, Kigali P.O. Box 3900, Rwanda; 3 Department of Microbiology and Parasitology, School of Medicine and Pharmacy, College of Medicine and Health Sciences, University of Rwanda, Kigali P.O. Box 3286, Rwanda; f.x.ndayambaje@ur.ac.rw (F.X.N.); j.b.munyemana@ur.ac.rw (J.B.M.); 4 School of Public Health, College of Medicine and Health Sciences, University of Rwanda, Kigali P.O. Box 3286, Rwanda; ndahindwa@nursph.org; 5 King Faisal Hospital, Kigali P.O Box 2534, Rwanda; fredrick.lukwago@kfhkigali.com (F.L.); lambert.ingabire@kfhkigali.com (L.I.); damas.dukundane@kfhkigali.com (D.D.);; 6 Clinton Health Access Initiative, Kigali P.O. Box 4062, Rwanda; fgambanga@clintonhealthaccess.org; 7 Repolicy Research Centre, Kigali P.O. Box 7584, Rwanda; p.gashema@sms.ed.ac.uk; 8 College of Medicine and Veterinary Medicine, The University of Edinburgh, Edinburgh EH8 9YL, UK; 9 International Centre for AIDS Care and Treatment Program, ICAP, Lusaka P.O. Box 34358, Zambia; td2581@cumc.columbia.edu

**Keywords:** antimicrobial resistance, antibiotic prescription, healthcare providers, infection prevention, Rwanda

## Abstract

Antimicrobial resistance (AMR) is a major public health threat linked to increased morbidity and mortality. It has the potential to return us to the pre-antibiotic era. Antimicrobial stewardship (AMS) programs are recognized as a key intervention to improve antimicrobial use and combat AMR. However, implementation of AMS remains limited in Africa, particularly in Rwanda. This study aimed to assess prescription practices, identify areas for improvement, and promote adherence to AMS principles. Conducted at King Faisal Hospital in Rwanda, this qualitative study used semi-structured interviews with eight participants until saturation was reached. The interviews were recorded, transcribed, and thematically analyzed, revealing four emerging themes. The first theme was on AMS activities that were working well based on availability of microbiology laboratory results and prescription guidelines as factors influencing antibiotic prescription adjustments. The second theme was related to challenges during the implementation of the AMS program, including the prescription of broad-spectrum antibiotics, limited local data on AMR patterns, and stock-outs of essential antibiotics. The third theme was on the importance of adhering to AMR management guidelines at KFH. The last emerged on recommendations from participants centered on regular training for healthcare workers, widespread dissemination of AMR findings across departments, and the enforcement of antibiotic restriction policies. These actions can improve prescription behaviors, upholding the highest standards of patient care, and strengthening the nascent AMS program.

## 1. Introduction

Antimicrobial resistance (AMR) has emerged as a significant global health threat. This phenomenon renders once-effective antibiotics ineffective, posing a significant challenge to the management of infectious diseases [1]. Some of the contributors to the AMR issue include physicians, patients, pharmacists, and health authorities [2]. In response, healthcare facilities have implemented antimicrobial stewardship (AMS) programs as a vital strategy to combat AMR [3,4]. These programs aim to optimize the use of antimicrobials through a focus on interventions such as minimizing antimicrobial therapy duration, utilizing rapid diagnostic tests, and implementing clinical decision support tools [3]. AMS programs have been successful in reducing antimicrobial use and the spread of resistant pathogens [5]. Discouraging empirical treatment by relying on microbiology investigations and restricting the duration of antibiotic prescriptions are crucial components in reducing the burden of AMR pathogens [6]. Intervention targeting the improvement in antibiotic prescription should include clinicians as prescribers [7]. A review by Davey et al. [8] reported that reducing the prescription of antimicrobials significantly curbs AMR pathogens and improves microbiological and clinical outcomes. While AMS programs are widely recognized as a key strategy to combat AMR, some studies have highlighted a scarcity of robust evidence regarding their overall effectiveness [9]. Furthermore, data on AMS programs in low- and middle-income countries (LMICs) are scanty, especially in Africa [10]. To the best of our knowledge and research, there is no published literature on AMS programs in Rwanda. However, two initiatives are being initiated at the University Teaching Hospitals of Kigali (CHUK) and another one at the University Teaching Hospitals of Butare (CHUB). A core strategy in successful AMS programs involves engaging prescribers at the point of prescribing antibiotics to promote informed decision making [11,12,13,14]. Recognizing the growing threat of AMR, King Faisal Hospital (KFH), a prominent healthcare institution in Rwanda, has taken a commendable step by initiating its own AMS program. We hypothesized that a considerable percentage of prescriptions of antibiotics are not laboratory-based and AMS principles are not adhered to. This qualitative study aimed at exploring prescription practices to shed light on the experiences, perceptions, and challenges faced by healthcare providers and staff who work at KFH in adhering to AMS principles. This study is expected to allow the identification of gaps in the implementation of the AMS program and inform interventions and the way forward both at KFH and other facilities throughout the country.

## 2. Results

The saturation point was attained following interviews with eight (8) healthcare workers (HCWs), comprising four medical doctors, one pharmacist, one clinical nurse, one Infection Prevention and Control (IPC) officer, and one laboratory technician. Additional information about the informants can be found in Table 1 below and in Supplementary Table S1: Consolidated criteria for reporting qualitative studies (COREQ). During analysis, four main themes were identified and the associated codes, descriptions, and summaries of the themes are highlighted below.

### 2.1. AMS Activities That Were Working Well

All interviewed healthcare workers (HCWs) identified aspects of King Faisal Hospital’s (KFH) AMS program that were functioning well. These positive aspects fell into several key sub-themes.

#### 2.1.1. Laboratory Utilization: HCWs across Various Departments (ICU, Pediatrics, and Laboratory) Emphasized the Importance of Laboratory Testing to Guide Antibiotic Selection—This Included Using Culture Results to Adjust Initial Empiric Therapy and following Established Protocols for Requesting and Interpreting Tests

“For us when a patient comes to ICU, we check their diagnosis and if there is a corresponding protocol, especially international, for example, if someone has sepsis, you assess the score then with this you can tell which medicine to give, does he need an antibiotic or not, so we base on the patient’s assessment. You can guess which is the likely microbe, if it is resistant or not and then, so you do laboratory, tests and start antibiotics if you find the pertinent need. Then, you can stop after you get the antibiogram, so I can say that here at King Faisal, there is no problem. I have visited the laboratory, and I didn’t find any problem”.

“ICU Doctor”—KFH/HCW/006-

#### 2.1.2. Communication and Guidelines: Several HCWs Mentioned Clear Communication Protocols within the Hospital Regarding AMS—This Included Timely Reporting of Laboratory Results and Adherence to National Guidelines for Antibiotic Use

“Most of the time: when the patient comes with fever, we check sensitivity, but when the patient is severely ill, she is put-on broad-spectrum antibiotic then waits for 2–3 days. Then, adjust antibiotic after getting laboratory results”.

“Nurse in ICU”—KFH/HCW/003-

“Well, here in hospital, there are guidelines regarding how we communicate within the Hospital. When we receive something like a blood culture or a swab, we follow those guidelines. If, for instance, a sample like a blood culture is growing, we have to communicate with the doctor. We inform them that the bacteria that’s growing is either gram-negative or gram-positive, and we provide them with a preliminary report. Then, we tell them that after two days, when checking the system, there will be the final result”.

“Laboratory technician”—KFH/HCW/004-

#### 2.1.3. Infection Prevention and Control (IPC): The IPC Department Was Highlighted as a Key Player in the AMS Program—HCWs Described Routine Practices Such as Handwashing Education, Environmental Cleaning, and Equipment Maintenance to Prevent the Spread of Resistant Pathogens

“All of us have taken this as a responsibility, And we do practice hand hygiene in the clinical areas. We practice the five moments of hand hygiene. We do monitor that and audit. And any gaps we find, we try to close them”.

“IPC Officer”—KFH/HCW/005-

### 2.2. Challenges with AMS

**Figure 1:**
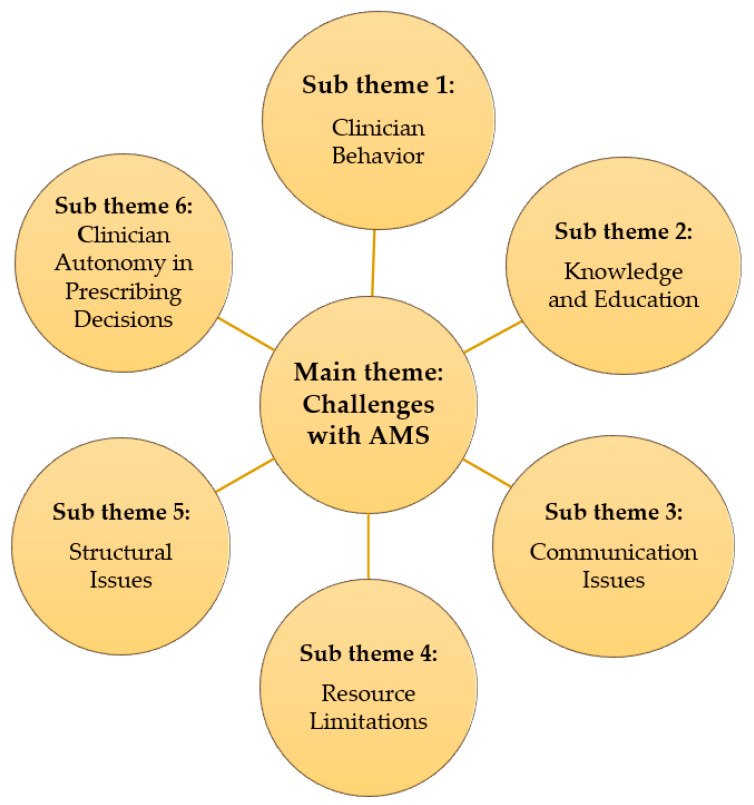
Challenges with AMS and associated sub-themes.

HCWs identified several challenges hindering optimal implementation of the AMS program at KFH. These challenges fell into several key sub-themes, which is also indicated in Figure 1 below.

#### 2.2.1. Clinician Behavior: Some Participants Reported Concerns about Antibiotic Overuse and Lack of Adherence to Established Protocols for Prescribing and Duration of Treatment

“I feel like sometimes, um, we, the health care personnel, we do abuse antibiotics, because sometimes we may order an antibiotic today, tomorrow we are changing it, another day we are changing it, and we don’t follow the protocols, not to start with and not to end with”.

“ICU Doctor”—KFH/HCW/006-

#### 2.2.2. Knowledge and Education: Several HCWs Highlighted a Lack of Training and Education on AMR among Staff, Particularly Regarding Interpretation and Utilization of Antimicrobial Resistance Data from the Laboratory

“Um, because it’s willing to assist us. It feels it knows the impact of the infections and it tries to support us. I can also request the Rwanda Biomedical Center (RBC) where you come from that whenever you have these, uh, trainings if you can always invite us so that we can also be updated on what is going on around in the world”.

“IPC Officer”—KFH/HCW/005-

#### 2.2.3. Communication Issues: Breakdowns in Communication between Personnel and Departments Were Identified as a Barrier to Timely Delivery and Utilization of Laboratory Results

“There’s no report, no communication that tells us anything, except for us to take the initiative to ask. At that moment, when we identify a germ that is resistant, we tend to call that we’ve encountered a patient with resistance to a certain antibiotic that is spreading rapidly, especially to this antibiotic. Then, we communicate that we have identified a germ that is resistant”.

“Laboratory technician”—KFH/HCW/004-

#### 2.2.4. Resource Limitations: Stockouts of Essential Antibiotics and Laboratory Supplies Were Reported as a Significant Challenge

“This is a challenge we usually encounter, some times we request for culture and sensitivity for UTI to which we usually prescribe quinolones but when the results come out you find they did not test the quinolones but instead they tested cephlosporins and you realize that has become a challenge to us and we do not know why in the laboratory they choose to test certain antibiotics and not others but there is a time back they told us that they had a stock out of some antibiotic discs though they usually have them most of the time”.

“Emergency Doctor”—KFH/HCW/007-

#### 2.2.5. Structural Issues: The Limited Functionality of the AMS Committee and Lack of a Dedicated Forum for Discussing AMR Issues Were Identified as Weaknesses in the Current System

Another key issue highlighted was the fact that KFH is a referral hospital and, sometimes, patients come and they are already prescribed a different antibiotic which is not effective or not the appropriate one. The issue of the clinician having final discretion on prescription was also raised as a challenge as it was mentioned that, sometimes, doctors do not keep up with guidelines and international recommendations. Sometimes, there are disagreements on the best antibiotics to go with even after laboratory reports, but the doctor (clinician) has the last say. Committees were mentioned but there is not much focus on AMR, the drug and therapeutic committee (DTC). And some respondents mentioned that they were part of the DTC but either had not attended or did not know when the meetings are held. An AMS committee was referenced but the respondent mentioned that it is still in its infancy stage and not yet very functional. Another challenge was the lack of a specific forum to discuss AMR issues. One respondent estimated that only 30% of antibiotic prescriptions were based on antibiogram. Another respondent said that there is no restriction on antibiotics. Whatever antibiotic he wishes, he prescribes. There is no preauthorization for certain antibiotics.

#### 2.2.6. Additionally, Some Participants Expressed Concerns about Clinician Autonomy in Prescribing Decisions, Suggesting That Some May Not Consistently follow National Guidelines or International Recommendations

“In the pediatric population, our approach to antimicrobial treatment aligns with the guidelines provided by the Ministry of Health or the Rwanda Biomedical Centre. These guidelines outline the use of first, second, and third-line antimicrobials for various conditions. When treating pediatric patients, our strategy for selecting antibiotics follows a similar path. We typically initiate treatment with first-line antimicrobials as empiric prophylactic therapy while awaiting a definitive diagnosis. For instance, in the case of newborns, our current practice recommends starting the initiation of treatment with ampicillin and gentamicin as first-line empiric therapy. However, it is concerning that we are observing high levels of resistance, with rates reportedly reaching up to 80% for these antibiotics. Despite this challenge, our adherence to guidelines mandates the initiation of treatment with ampicillin and gentamicin as the initial approach. If there is no improvement in the patient’s condition or if concerns arise regarding resistance, we then consider transitioning to second-line antimicrobials as per the guidelines”.

“Pediatrician Doctor”—KFH/HCW/002-

### 2.3. Use of Guidelines and Standard Protocols for AMR

HCWs brought up and referred to use of guidelines, policies, and protocols. HCWs mentioned different guidelines like International, National, Ministry of Health, and the Rwanda Biomedical Centre (RBC) guidelines. However, HCWs appeared more familiar with hospital or department-based guidelines/protocols. Those guidelines are grouped into several key sub-themes.

#### 2.3.1. Compliance to Guidelines, Standards, or Protocols When It Comes to AMR: Most of Them Understand That if There Are Available Nationally Based Guidelines, Then That Can Help People to Adhere to the Local Hospital-Based Guidelines

“No! When we conduct testing, we need to adhere to established standards. We adhere to these standards and then send the report. It’s up to them to take the initiative to provide the medicine because often, you can see in the treatment that it may need to start. The patient can start the treatment, the patient arrives with symptoms that match the sample taken to the laboratory, and then they start the antibiotic to save time because microbiology results can take like two to three days. So, these days, the patient needs to start the antibiotic. Therefore, when we conduct testing, we follow established standards and I third of them also mentioned antimicrobial stewardship policy”.

“Laboratory technician”—KFH/HCW/004-

#### 2.3.2. Departmental Guidelines: Infection Surveillance in ICU Guidelines Outline the Protocol for Any Patient Who Is Admitted in ICU; They Have to Perform a Blood Culture Sensitivity

“Um, we have a policy on infection surveillance whereby every patient who comes into our hospital is expected to have a blood culture done on them. But because of our insurance, it becomes a little bit difficult. But where we have found it’s not very difficult is for our patients who are admitted in ICU. Any patient who is admitted in ICU, they have to do a blood culture sensitivity so that they are able to know what infection the patient has. They came with it from home, or they acquired it from a hospital? And any devices they come with, like the urinary catheters, the IV cannulas, we do remove them from accident and emergency, before they go into the unit. So that we start again and fix ours”.

“ICU Doctor”—KFH/HCW/006-

### 2.4. Recommendations for How AMS Program Can Be Improved at KFH

**Figure 2:**
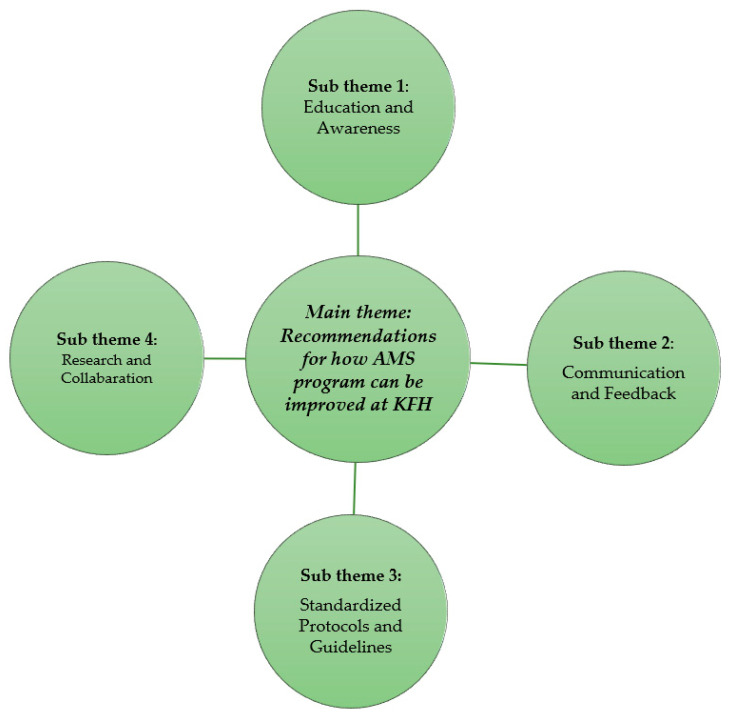
Recommendations for how AMS program can be improved at KFH and associated sub-themes.

Participants provided several recommendations to strengthen the AMS program at KFH. These recommendations fell into several key sub-themes and are indicated in Figure 2 below.

#### 2.4.1. Education and Awareness: Participants Emphasized the Need for Ongoing Training and Education for All Staff on AMR, Including Interpretation of Antimicrobial Resistance Data and Appropriate Antibiotic Prescribing Practices

“Way forward! Except cases we discuss in session when we see an infection; Also when we send samples (environmental swabs) to the lab, when results are releases, we discuss about the results. For instance, last time in July and August we had training on antibiograms and antibiotic stewardship which can be continuous for smooth running of AMS”.

“Pediatrician Doctor”—KFH/HCW/002-

#### 2.4.2. Communication and Feedback: Improved Communication between Laboratory Personnel, Pharmacist, and Clinicians Was Identified as Essential—This Includes Timely Delivery of Test Results and Mechanisms for Laboratory Staff to Understand How Clinicians Are Utilizing This Information

“In our collaboration as a pharmacy, when a doctor prescribes an antibiotic, we often find that certain tests are necessary to support their decision. In such cases, the pharmacy contacts the lab to confirm whether these tests have been conducted. This demonstrates collaboration between the pharmacy and the lab/microbiology department which is recommended for good practice”.

“Pharmacist”—KFH/HCW/001-

“As I am the manager, every morning I am supposed to check into the system to know newly admitted patients, sometimes they are like 14 or 13. I just go there as I have the history and the clinician is very busy or we may have critical cases (severely ill patients). So, I have my responsibility to go and check. When I get results, I inform the clinician or sometimes the lab can call the clinician but often we are the ones to check what we sent if results are available for the purpose of guiding clinical decisions”.

“Pediatrician Doctor”—KFH/HCW/002-

#### 2.4.3. Standardized Protocols and Guidelines: A Call Was Made for Clearer and More Consistent Use of National and Hospital-Based Guidelines for Antibiotic Prescribing

“Considering the recent trend of antimicrobial resistance among our patients, for instance, some individuals believe that only certain antibiotics can cure them, leading them to prescribe ceftriaxone yet it would have been more prudent to start with Amoxicillin. I believe it is essential to initiate treatment with first-line or lower-class antibiotics rather than higher classes. Additionally, I would recommend that doctors use antibiograms more frequently to guide their prescribing practices”.

“Pharmacist”—KFH/HCW/001-

#### 2.4.4. Research and Collaboration: Several Participants Highlighted the Value of Conducting Local Research on AMR Patterns and Fostering Collaboration between Clinicians and Researchers to Inform Best Practices

“Yes, of course, Rwanda is ahead in many things there is no reason to limit ourselves. I believe these things of antibiograms, and antibiotics are not complex, you must train people. I remember when we were in Butare we could see laboratory technicians doing that work and then sharing guidance with clinicians. So, it means that we cannot wait to have specialists everywhere for admitted patients for us to achieve these targets. We all treat patients, and all patients deserve the best care which means no harmful prescriptions. So, I say that I wish that research can be promoted, collaboration of clinicians and researchers in the center like RBC, so we can analyze our own data draw specific conclusions. The problem is that clinicians do not have time for writing grants and they may not have the right skills for that. That is the advice I can give”.

“IPC Officer”—KFH/HCW/005-

## 3. Discussion

This qualitative study revealed several strengths of the AMS program at KFH. One positive finding is the understanding of AMR by the healthcare workers and the role played by the microbiology laboratory to determine effective antibiotics important for adjusting prescriptions. This finding aligns, to some extent, with studies conducted among physicians and nurses in Uganda [15], Gabon [16], Nigeria [17], South Africa [18], and Spain [7]. However, it is important to acknowledge that limited knowledge and awareness of AMS programs and AMR have also been documented in healthcare settings in Saudi Arabia [19]. This disparity in knowledge levels might be partially attributed to the relatively recent introduction of AMS programs in many LMICs [10]. These findings highlight the critical need for regular and ongoing educational programs to bolster healthcare workers’ understanding of both AMR and AMS principles.

Interview participants emphasized the importance of relying on microbiology laboratory results while prescribing antibiotics or adjusting them. This concurs with the study conducted in Norway [12]. It has been shown that reliance on microbiology laboratory results discourages empirical treatment and can reduce the duration of antimicrobial therapy, which are crucial in curbing AMR trends [10,20,21]. Morency-Potvin et al. [22] showed that clinical microbiologists are pillars in AMS programs as they produce cumulative antimicrobial susceptibility reports used as evidence of AMR to alert surveillance systems. The nascent AMS program at KFH should strengthen the microbiology laboratory and its staff to provide regular reports and meetings to share findings of pathogens with increased resistance.

This study also revealed that KFH staff demonstrated awareness of the importance of hand hygiene in infection prevention and control (IPC). Improper hygienic practices lead to infections that promote use of antimicrobials known as a driver of AMR [23,24]. Additionally, participants mentioned other practices such as routine fumigation and environmental swabbing that contribute to maintaining a hygienic hospital environment and mitigating the spread of resistant pathogens. Previous studies highlighted water, sanitation, hygiene, and IPC among important weapons to reduce the AMR burden [23,25]. While awareness of these practices is positive, it is essential to go beyond mere knowledge. Regular reinforcement through morning staff meetings and patient education initiatives can promote behavior change and lead to long-term improvements in IPC practices.

The laboratory key informants mentioned that the laboratory staff communicate preliminary results to the clinicians. The laboratory staff highlighted adhering to the set turn-around time (TAT). KFH also uses an electronic system, which significantly improved the reporting period when compared to the old paper-based system. This allows quick access to results immediately after being generated. However, microbiologists should keep the practice of calling the requesting department for critical results. It is also important to think about introducing an alert system that can send a text message to the clinicians. Despite these successes, the interviews also revealed challenges associated with the AMS program. Some participants mentioned instances where patients were initially placed on broad-spectrum antibiotics, with adjustments made only after receiving laboratory results. Additionally, a clinician expressed concern about the lack of restrictions on certain antibiotics. The World Health Organization (WHO) has developed the Access, Watch, Reserve (AWaRe) classification system for antibiotics, aiming to promote the judicious use of these medications. This system categorizes antibiotics based on their importance for public health. The goal is to encourage the use of Access category antibiotics for at least 60% of prescriptions, while limiting the use of those in the Watch and Reserve categories [26]. A previous study demonstrated that the adoption of a computerized prescriber authorization system for certain antibiotics led to a decrease in broad-spectrum antibiotic prescriptions and a corresponding increase in narrow-spectrum antibiotic prescriptions [27]. However, the effectiveness of antibiotic restriction policies in reducing AMR remains a topic of ongoing debate. KFH is advised to revise the AMR trend and set a list of antimicrobials that will be placed in the watch and reserve categories. Also, conditions to grant such antimicrobials should be clear and adhered to. Moreover, stockouts of essential antibiotics and laboratory supplies, communication breakdowns, and delays in receiving laboratory reports were identified as impediments to effective AMS implementation. The lack of clarity on utilizing available AMR data and varying antibiotic prescription practices among clinicians further exacerbate the challenge [28]. Similar challenges have been listed to be common in several LMICs [20,29,30]. It is crucial for KFH management to address raised challenges to ensure the sustainability of the initiated AMS program. The AMS committee should serve as a link between the laboratory and clinicians by ensuring that AMR results from the laboratory are regularly reviewed. Also, regular training would help to improve awareness on AMR and AMS of KFH personnel. This study had some limitations. These include the small number of healthcare workers interviewed, which could not be representative of the KFH staff and one clinician from internal medicine missed two planned interview appointments and limits the generalizability of the findings at the national level. Also, respondents were proposed by the KFH Director of Research who selected experienced staff and this could bias the study findings.

## 4. Materials and Methods

### 4.1. Study Design

This was an exploratory qualitative study. It involved an in-depth interview with key informant healthcare providers and allied health staff from different departments at King Faisal Hospital (KFH). A semi-structured interview guide was used to determine factors influencing the antimicrobial prescription at the hospital. The exploratory qualitative study was chosen as it assisted researchers in exploring views from different key informants on a set of predetermined questions to face-to-face interviews.

### 4.2. Study Site and Selection

This study was conducted at the KFH located in Kigali City, Rwanda, from 6th to 14th December 2023 and ethical approval was obtained from KFH with reference number KFH/2023/102/IRB. In 2023, KFH was upgraded to the level of being a University Teaching Hospital. KFH was chosen based on the previous study that established baseline data on AMR in Rwanda and the fact that its AMS program is nascent.

### 4.3. Study Interview Guide Development

The study team developed an interview guide based on the study objectives and the literature. This study was conducted to assess practices related to AMR and AMS programs at KFH. The guide included open-ended questions on awareness of AMR, communication of laboratory and clinicians, antibiotic prescription, antimicrobial stewardship, IPC, etc. Then, face-to-face interviews were organized and conducted physically at KFH with eight (8) key informants.

### 4.4. Recruitment of Participants

Participants were purposively selected from each department including (i) emergency, (ii) intensive care unit (ICU), (iii) neonatal intensive care unit (NICU), (iv) the laboratory, (v) pharmacy, (vi) pediatrics, (vii) surgical ward, (viii) IPC nurse, and (ix) internal medicine. Key informants were identified through a mapping exercise with the help of the Director of Research at KFH. Participants included an emergency physician, an internal medicine physician also trained on critical care, a consultant neonatal pediatrician, a pediatrician, a critical care nurse, a pharmacist, the laboratory manager, and one IPC nurse. Two consultants (internal medicine and surgery) did not receive time for the interview.

### 4.5. Key Informants’ Interviews (KIIs)

Face-to-face interviews with key informants were conducted by trained and experienced researchers. NG holds a Ph.D in Clinical Microbiology and works at the Rwanda Biomedical Centre, VD is a medical doctor with a Masters in Public Health and works at the University of Rwanda, College of Medicine and Health Sciences, and MG also holds a Masters in Public Health and works at the Rwanda Biomedical Centre. Interviews were conducted in a quiet place within KFH premises. Before the interview, verbal consent was sought from the interviewee to record the conversation and participants were informed that the duration of the interview would last between 30 and 45 min. Interviewers were informed to respond in either English or Kinyarwanda and interviews were recorded. The interview was conducted in either Kinyarwanda or English or a mix of both languages depending on the choice of the interviewee. All records were translated into English by a qualified interpreter.

### 4.6. Data Analysis

Data were analyzed using a thematic analysis approach. Recorded KIIs were transcribed and translated from Kinyarwanda to English. All transcripts were cross-checked to ensure completeness of data by the interviewers and researchers. All transcripts were read through and codes were generated by a separate researcher. Dedoose software was used to create codes and analyze the data. Responses with similar codes were recategorized under a unifying theme. After data were coded in Dedoose, they were then manually organized into themes. Themes were then interpreted for their descriptive meaning. Descriptive quotes representing key themes were identified.

## 5. Conclusions

These findings underscore the factors influencing prescription practices at KFH. They also highlight the role played by the AMS program in improving adherence to AMS principles among healthcare professionals at KFH. Educational initiatives to strengthen knowledge of healthcare workers on AMR, widespread dissemination of AMR findings across departments, and the enforcement of antibiotic restriction policies were mentioned by interviewees as crucial for the rational use of antimicrobials.

## Figures and Tables

**Table 1:** Key informants’ details, function, and years of experience in the services.

Study Number	Healthcare Workers Function	Period in Service
KFH/HCW/001	Pharmacist	7 years
KFH/HCW/002	Pediatrician Doctor	12 years
KFH/HCW/003	Nurse in ICU	15 years
KFH/HCW/004	Laboratory technician	12 years
KFH/HCW/005	IPC Officer	18 years
KFH/HCW/006	ICU Doctor	11 years
KFH/HCW/007	Emergency Doctor	8 years
KFH/HCW/008	General Practitioner in the emergency Department	14 years

## Data Availability

The raw data (transcripts) are available upon requested.

## Supplementary Materials

The following supporting information can be downloaded at: https://www.mdpi.com/article/10.3390/antibiotics13060548/s1, Supplementary Table S1: Consolidated criteria for reporting qualitative studies (COREQ): 32-item checklist. Reference [31] is cited in the Supplementary Materials.
