# Supplementary material for: Exploring Prescription Practices: Insights from an Antimicrobial Stewardship Program at a Tertiary Healthcare Facility, Rwanda
Source: Antibiotics (Basel). 2024 Jun 12;13(6):548. doi: 10.3390/antibiotics13060548 (PMC11200619; doi:10.3390/antibiotics13060548)
Supplement: Supplementary file 1 [file antibiotics-13-00548-s001.zip › antibiotics-3023114-supplementary.pdf]

Manuscript: Exploring Prescription Practices: Insights from an Antimicrobial Stewardship Program at a Tertiary Healthcare Facility, Rwanda

Supplementary Table S1. Consolidated criteria for reporting qualitative studies (COREQ): 32-item checklist. Developed from: Tong, 2007 [31].

**No. Item**	**Guide questions/description**	**Reported on Page #**
Domain 1: Research team and reflexivity		
Personal Characteristics		
1. Interviewer/facilitator	Which author/s conducted the interview or focus group?	Page 9 / Paragraph 6
2. Credentials	What were the researcher's credentials? E.g. PhD, MD	Page 9 / Paragraph 6
3. Occupation	What was their occupation at the time of the study?	Page 9 / Paragraph 6
4. Gender	Was the researcher male or female?	Page 9/ _aragraph 6
5. Experience and training	What experience or training did the researcher have?	Not reported on page, but researcher possess vast experience in AMR Program
Relationship with participants		
6. Relationship established	Was a relationship established prior to study commencement?	Not reported on page
7. Participant knowledge of the interviewer	What did the participants know about the researcher? e.g. personal goals, reasons for doing the research	Page 9/ Paragraph 5 Participants were briefed by Director of Research at KFH the purpose of study and ethical approval was granted from IRB at KFH and participation was voluntarily and informed consent was obtained from them
8. Interviewer characteristics	What characteristics were reported about the interviewer/facilitator? e.g. Bias, assumptions, reasons and interests in the research topic	Interview guide is available in the supplementary files S1 Page 9/ Paragraph 4

Domain 2: study design		
Theoretical framework		
9. Methodological orientation and Theory	What methodological orientation was stated to underpin the study? e.g. grounded theory, discourse analysis, ethnography, phenomenology, content analysis	Page 9–10
Participant selection		
10. Sampling	How were participants selected? e.g. purposive, convenience, consecutive, snowball	Page 9/ Paragraph 5
11. Method of approach	How were participants approached? e.g. face-to-face, telephone, mail, email	Page 9 / Paragraph 2
12. Sample size	How many participants were in the study?	Page 9/ Paragraph 4
13. Non-participation	How many people refused to participate or dropped out? Reasons?	Two consultants: Page 9/ Paragraph 5
Setting		
14. Setting of data collection	Where was the data collected? e.g. home, clinic, workplace	Page 9/ Paragraph 6
15. Presence of non-participants	Was anyone else present besides the participants and researchers?	No
16. Description of sample	What are the important characteristics of the sample? e.g. demographic data, date	Page 2 / Table 1
Data collection		
17. Interview guide	Were questions, prompts, guides provided by the authors? Was it pilot tested?	Yes, questions were provided by authors and pilot was done / Page 9 / Paragraph 4
18. Repeat interviews	Were repeat inter views carried out? If yes, how many?	**No**
19. Audio/visual recording	Did the research use audio or visual recording to collect the data?	Yes. Page 9, paragraph 6
20. Field notes	Were field notes made during and/or after the interview or focus group?	Field notes was done during interview
21. Duration	What was the duration of the interviews or focus group?	Page 9 / Paragraph 6
22. Data saturation	Was data saturation discussed?	Page 2 / Paragraph 2
23. Transcripts returned	Were transcripts returned to participants for comment and/or correction?	No
Domain 3: analysis and		

findings		
Data analysis		
24. Number of data coders	How many data coders coded the data?	Four authors
25. Description of the coding tree	Did authors provide a description of the coding tree?	Page 9 / Paragraph 7
26. Derivation of themes	Were themes identified in advance or derived from the data?	Themes were derived from the data
27. Software	What software, if applicable, was used to manage the data?	Page 9 / Dedoose / Paragraph 6
28. Participant checking	Did participants provide feedback on the findings?	Yes
Reporting		
29. Quotations presented	Were participant quotations presented to illustrate the themes/findings? Was each quotation identified? e.g. participant number	Under every theme participant quotation were used as well as Participants function at health facility / Study number
30. Data and findings consistent	Was there consistency between the data presented and the findings?	Yes
31. Clarity of major themes	Were major themes clearly presented in the findings?	Yes, Page 2–7 / All themes were clearly presented
32. Clarity of minor themes	Is there a description of diverse cases or discussion of minor themes?	We discussed major themes and associated subthemes in the main text
